# Artificial intelligence in healthcare text processing: a review applied to named entity recognition

**DOI:** 10.3389/frai.2025.1584203

**Published:** 2025-07-07

**Authors:** Samuel Santana de Almeida, Raphael Silva Fontes, Luca Pareja Credidio Freire Alves, Methanias Colaço Júnior, Gleyson José Pinheiro Caldeira Silva, Lyane Ramalho Cortez, Antonio Higor Freire de Morais, Guilherme Medeiros Machado, Hugo Gonçalo Oliveira, Aliete Cunha-Oliveira, João Paulo Queiroz dos Santos, Ricardo Alexsandro de Medeiros Valentim

**Affiliations:** ^1^Postgraduate Program in Computer Science (PROCC), Federal University of Sergipe, São Cristóvão, Brazil; ^2^Center for Innovation and Advanced Technology (NAVI), Federal Institute of Rio Grande do Norte, Natal, Brazil; ^3^Laboratory for Technological Innovation in Health (LAIS), Onofre Lopes University Hospital, Federal University of Rio Grande do Norte, Natal, Brazil; ^4^ECE Paris Engineering School, Paris, France; ^5^Department of Informatics Engineering (DEI), University of Coimbra, Coimbra, Portugal; ^6^Nursing School of Coimbra (ESEnfC), Coimbra, Portugal

**Keywords:** named entity recognition (NER), health texts, BERT model, advanced language models, ChatGPT, SUS

## Abstract

**Context:**

Traditional methods such as rule-based systems, word embeddings (e.g. Word2Vec, GloVe) and sequence tagging models such as CRFs and HMMs have difficulty capturing the complex and nuanced context of medical texts, leading to low precision and inflexibility. These methods also struggle with the inherent variability of medical language and often require large and difficult-to-obtain labeled datasets.

**Objective:**

We examine the growing importance of Named Entity Recognition (NER) in the analysis of healthcare texts. NER, a fundamental technique in Natural Language Processing (NLP), automatically identifies and categorizes named entities in the text, such as names of people and organizations, in medical texts, medical conditions and drug names. This facilitates better information retrieval, personalized medicine approaches and clinical decision support systems.

**Methods:**

A systematic mapping was carried out that focused on advanced language models, specifically transformation-based models such as BERT. These models are known for capturing complex semantic dependencies and linguistic nuances, which are crucial for accurate processing of medical texts. Transformation architectures, unlike traditional techniques such as CNNs and RNNs, are better suited to dealing with the contextual and semantic nature of medical texts due to their ability to manage long sequences and the need for high precision.

**Results:**

The results indicate that transformation-based models, in particular BERT and its specialized variants (e.g. ClinicalBERT), consistently demonstrate high performance on NER tasks, with F1 scores often exceeding 97%, outperforming traditional and hybrid methods. When examining the geographical distribution of contributions, the research identifies a significant contribution from China, followed by the United States. These findings have crucial implications for the integration of NER technologies into the Brazilian National Health System (SUS).

**Conclusion:**

This systematic review contributes to the advancement of NER in health texts by evaluating methods, showing results and highlighting the wider implications for the field. The article is systematically structured into the following sections: Methodology, Bibliometric analysis, Results and discussion, Threats to validity, Future work and Conclusion. This systematic organization provides a comprehensive review of the research, its impact and future directions, highlighting the importance of keeping up to date with advances in the field to increase the relevance of NER applications in healthcare.

## 1 Introduction

The right to health is universally recognized as a fundamental element of human rights (PENSESUS—Portal de Informações de Saúde Pública da Fiocruz, 2023a). This principle is enshrined in the 1948 Universal Declaration of Human Rights, Article XXV, which states that every human being has the right to a standard of living adequate for health and well-being, including medical care and necessary social services. It is demonstrated a milestone in civilizational progress and the expansion of care for human life, prompting reflections on the implementation of effective health systems to ensure equitable access for the population, contributing to the health and well-being of all.

In Brazil, a pivotal event in the health domain was the creation of SUS in 1988. Regulated by Laws No. 8080 and 8142 of 1990, SUS represents a significant framework to provide universal and equal medical care to all citizens (PENSESUS—Portal de Informações de Saúde Pública da Fiocruz, 2023b). However, the pursuit of the full realization of the right to health faces substantial challenges, including funding issues (Mendes and Marques, 2009), regional inequalities, and the need for a broader approach that goes beyond curing diseases and considers prevention and health promotion (Brito-Silva et al., 2012).

Artificial intelligence (AI) has the potential to address various scenarios, including health, the application of techniques related to natural language processing and machine learning proves to be a promising starting point for developing increasingly complex tools in the health domain (Milne-Ives et al., 2020).

In this context, NLP can be highlighted as a driving force for technological innovation in this niche (Turchioe et al., 2022). This field aims to train machines to understand, interpret, and generate text in a way similar to humans (McCarthy et al., 2006). A common task in the scope of NLP is text classification, where the goal is to assign categories of a predefined set to documents or sentences, based on their content. More complex tasks involve labelling each token in a sequence according to the context. NER is a popular NLP task that falls on the previous description, as it aims at identifying and classifying named entities in a text (Li et al., 2020), into as names of people, locations, organizations, dates, and, depending on the domain, other key elements. This enhances semantic understanding, improving information retrieval, personalization, recommendation systems, and contextual sentiment analysis.

The objective of this work is to analyze the state of the art of AI in the realm of textual news published on the internet, presenting a systematic mapping of the literature to identify the most effective techniques for NER related to healthcare.

The application of these more advanced techniques can significantly improve the accuracy and contextualization of textual analysis in healthcare. Traditional models, such as those based on rules, word embeddings or sequence classification models like the hidden Markov model (HMM), have significant limitations (Xing et al., 2014; Feng et al., 2006). They cannot effectively capture the complexity and context of medical texts, which results in low accuracy, lack of contextual understanding and inflexibility.

These models struggle to handle the variability of medical texts, often requiring large amounts of labeled data, which is difficult to obtain. Furthermore, their rigidity prevents adaptation to new information or variations in the data. For example, rule-based models are effective only in specific cases and do not adapt well to new data, while techniques like Word2Vec and GloVe fail to capture the dynamic context necessary for in-depth analysis (Kulshretha and Lodha, 2023).

This integration between SUS promises significant advances in both computing and healthcare fields, aligning with the fundamental principles of SUS, which aim to provide universal and equitable access to the health system, the use of NER in the SUS allows for the automatic organization of medical information, improvement of disease surveillance, optimization of hospital resource management, integration of data from different systems and support of evidence-based decisions. This results in faster care, more efficient public policies and better quality of care for the population. After the review, the aim is to contribute to the area of computing with the development of artificial intelligence techniques, applying artificial intelligence to the extraction of data within large sets of information, favoring more accessible and inclusive computing. The remaining sections of this article are structured as follows:

Section 2—Methodology: Readers will get an elaborate description of the methods and techniques employed, research setting, background on how the research was done and where the data was collected and analyzed, along with any ethical considerations or methodological issues they may have.
Section 3—Results and discussion: Readers will get access to the content of the research findings, as well as an analysis of their advantages/disadvantages against the framework as stated in the study and literature, relevant so that readers have understood the exact findings, their connections with the literature and any implications or conclusions.
Section 4—Narrative synthesis: Provides an integrated and unified presentation of the main points of the study outlined in a direct, clear and concise manner.
Section 5—Threats to validity: Those that we consider it would be prudent to raise in discussion with readers as implications for the validity of the results, allowing for critical thinking regarding our findings.
Section 6—Concluding remarks: On the previous results and discussions providing a comprehensive perception of the theoretical and practical relevance of conducting this study some possible directions for future research.
Section 7—Future work: This section gives the Readers some insights into potential/important aspects that should be examined further, following the results of the study, this will serve as a good motivation for future research.

## 2 Methodology

With the aim of analyzing and evaluating NLP techniques for named entity extraction used in the healthcare domain, the systematic literature mapping (SLM) method was initially chosen. This process began on April 25, 2024. Systematic Literature Mapping emerges as a valuable tool when the demand is not for in-depth answers to specific questions but rather for obtaining a comprehensive and holistic view of a particular domain or area of knowledge (Kitchenham and Charters, 2007). Unlike more specific approaches, the primary goal of systematic mapping is to understand and systematically organize the existing body of literature. To achieve this, it is necessary to define the research orientation, the search strategy, and the criteria for article selection.

Table 1 illustrates the (Population, Intervention, Control, Outcome) PICO model used in this study, we used the PICO framework to formulate research questions. The idea of structuring clinical questions into four components was originally proposed by Richardson et al. (1995). When formulating a research question using the PICO model, researchers can structure their investigations clearly and specifically (Santos et al., 2007). This approach is useful for delimiting the research scope, identifying key variables, and facilitating the search for relevant evidence in the literature.

**Table 1:** Structured questions in PICO format.

Acronym	Category	Description
P	Population	Publications that address the extraction of named entities in health documents.
I	Intervention	Context of bidirectional language pre-training techniques, transformers or large language models used in the extraction of named entities in health-related documents.
C	Control	Conventional approaches that do not utilize these advanced language pre-training techniques for named entity extraction in healthcare documents.
O	Outcomes	The effectiveness of named entity extraction and the level of automation achieved in extracting entities from medical records.

Based on the definition of PICO, the review was guided by the following research questions.

- Q1: What are the main techniques used?
- Q2: Which specific techniques perform best and worst (Assessment basis language and Learning Type)?
- Q3: How are publications related to the use of bidirectional language pre-training techniques, Transformers, or LLMs in the extraction of named entities from health-related documents distributed across years?
- Q4: Which countries have contributed most significantly to publications in the context of bidirectional language pre-training techniques, Transformers, or large language models used in the extraction of named entities from health-related documents?

Given that, Population (P): The target group—health-related documents and named entity extraction. Intervention (I): The technique evaluated—the use bidirectional language pre-training techniques, Transformers, and large language models (LLMS). Control (C): The alternative for comparison -model architectures or learning types. Outcome (O): The main results—model performance (e.g., F1 score, Recall, Precision) and the distribution of publications or contributions by country.

The research questions map these elements as follows:

Q1 addresses the main techniques used (I) for NER on health-related documents (P).
Q2 focuses on which techniques perform best or worst, considering language and learning type as comparative (C) or control factors, and evaluates their results (O).
Q3 examines the distribution (O) of publications (P, I) over time.
Q4 investigates which countries (C) contributed most to the literature (O) on these techniques (I, P).

### 2.1 Search and selection strategy

To execute the search for articles, databases responsible for publishing the leading journals in the field of Computer Science were selected. These include SCOPUS, IEEE Xplore, ACM, Web of Science, Springer, and ScienceDirect. We wanted to cover all the databases that index the main articles in the area of computing, a key area for NER in health. For the search process, filtering tools provided by each database were utilized, focusing on title, abstract, and keywords. Access to the databases was granted through the CAPES journal portal (CAPES, 2021) using institutional subscriptions, with no restrictions on article access.

We start the list with Scopus, a large database that contains many important journals in the sciences and is reliable in all kinds of fields. IEEE Xplore, managed by IEEE (The Eminent Institute of Electrical Technology) and related sciences, contributes to the basis of this research, both platforms to a large extent. Another incredible resource is the Association for Computing Machinery, the ACM Digital Library being one of the largest electronic libraries in the field of computing and information technology. With a wide range of journals, conferences and technical papers, the ACM Digital Library is very important for researchers to have the latest first-class information on computer science, AI and SD/Software Development readily available.

Contributing to the research was the Web of Science database from Clarivate Analytics. This platform is widely known for its rigor and quality in indexing high-quality journals in various domains. It offers comprehensive data on citations and the influence of scientific publications on the Web of Science through cutting-edge analytical tools to help researchers assess research and trends in scientific territories. Elsevier and with its complementary ScienceDirect package and its extensive database of peer-reviewed articles, the platform includes health, life sciences, physical sciences and engineering has also used its access to good quality content to support the sharing of scientific knowledge and encourage all aspects of academic activity in all fields.

Last but not least, one of the best scientific publishers, Springer also occupies a significant position in world academia. Multiple fields of publication (computer science, artificial intelligence, etc.). Springer’s engineering sections have many articles, books and conference proceedings. Springer is one of the recognized platforms for the advancement and dissemination of new scientific discoveries, offers researchers highly appropriate and academically useful thematic resources.

Combining the resources offered by Scopus,^1^ IEEE Xplore,^2^ ACM Digital Library,^3^ Web of Science,^4^ Springer,^5^ and ScienceDirect,^6^ these platforms each with their own specializations and strengths is fundamental for building a solid and reliable foundation for scientific research, promoting continuous progress in their respective fields, and significantly contributing to the advancement of global knowledge. To conduct the search in digital databases, a search string composed of English terms and synonyms related to named entity recognition in health-related texts was defined. The terms were identified based on the roles defined in the PICO model, described in Table 1. Table 2 presents the terms adapted for optimal string utilization, and the subsequently refined terms are shown in Table 3.

**Table 2:** PICO model categories and terms identified for literature search.

Category	Keywords
Population	health*, clinical*, medical*
Intervention	LLM*, Large Language Model*, BERT*, Bidirectional Encoder Representations from Transformers, NER, Named Entity Recognition
Control	CNN, RNN
Outcomes	performance metrics, accuracy assessment, model evaluation

*It indicates that the keyword matches any term that begins with the stem specified before the asterisk.

**Table 3:** String after refinement.

Population	Intervention	Outcomes
Health	BERT	NER
	LLMs	

The reason for not using sequential models such as Convolutional Neural Networks and Recurrent Neural Networks (RNNs) on the task of Name Entity Recognition in medical texts, is that CNNs and RNNs are powerful models in tasks that involve sequence focus, including examples of patterns in images (Rodrigues, 2018) and temporary series, respectively, but NER requires a concept of contextual and semantics between deep words. BERT and LLMs were chosen on the merits of their ability to capture long-range dependencies and represent the nuances of natural language in a deeper way, which is crucial for identifying entities in medical texts; BERT, for example, is bidirectional, i.e., it considers the context to the left and right of the word.

Based on the considerations above, the following search string was developed, along with specific strings tailored for each database:

*STR01* (llm OR “large language model” OR BERT OR “Bidirectional Encoder Representations from Transformer”) AND (NER OR “Named Entity Recognition”) AND (health OR medical OR clinical) Database-Specific Search Strings.
*Scopus* (ABS(llm OR “Large Language Model” OR BERT OR “Bidirectional Encoder Representations from Transformer”) AND ABS(NER OR “Named Entity Recognition”) AND ABS(health OR medical OR clinical)).
*IEEE Xplore Digital Library* (“Abstract”:llm OR “Abstract”:“Large Language Model” OR “Abstract”: BERT OR “Abstract”:“Bidirectional Encoder Representations from Transformer”) AND (“Abstract”: NER OR “Abstract”:“Named Entity Recognition”) AND (“Abstract”:health OR “Abstract”:medical OR “Abstract”:clinical).
*ACM Digital Library* [[Abstract: llm] OR [Abstract: “Large Language Model”] OR [Abstract: BERT] OR [Abstract: “Bidirectional Encoder Representations from Transformer”]] AND [[Abstract: health] OR [Abstract: medical] OR [Abstract: clinical]] AND [[Abstract: NER] OR [Abstract: “Named Entity Recognition”]].
*Web of Science* (llm OR “Large Language Model” OR BERT OR “Bidirectional Encoder Representations from Transformer”) AND (NER OR “Named Entity Recognition”) AND (health OR medical OR clinical).
*ScienceDirect* (LLM OR “Large Language Model” OR BERT OR “Bidirectional Encoder Representations from Transformer”) AND (NER OR “Named Entity Recognition”) AND (health OR medical OR clinical).
*Springer* (ABS(llm OR “Large Language Model” OR BERT OR “Bidirectional Encoder Representations from Transformer”) AND ABS(NER OR “Named Entity Recognition”) AND ABS(health OR medical OR clinical)).

### 2.2 Source selection criteria

Inclusion and exclusion criteria are used to ensure that only appropriate studies or data are presented. Studies with a certain relevance/appropriateness to your research question and with minimal bias in study/information selection will be accepted.

Inclusion criteria: define the specific inclusion and data criteria that a study or data set must meet for inclusion in the current analysis. On the other hand, exclusion criteria state which studies or data need to be excluded due to lack of direct information or low quality of studies in the analysis, below we can see the criteria analyzed in the English language articles:

Inclusion criteria

- Recent articles (from 2019 onwards);
- Articles from scientific journals, either original or research articles.
  Exclusion criteria
- Duplicate articles;
- Secondary or tertiary studies;
- Works unrelated to the object of study;
- Works that did not detail practical experiments conducted to test their hypotheses.
  Quality evaluation criteria
- Does the study aim to specialize in a new model (fine-tuning)?

### 2.3 Information extraction strategy

Information extraction is a strategy that formulates a very detailed package of methods and techniques in which to identify and retrieve parts of the document text or unstructured data delivery. This strategy is common in the research fields of natural language processing and text mining and our goal is to extract valuable information that can be biased towards greatly enriching analyses.

The crucial initial step in the information extraction process is to define what the objectives and target information to be extracted are. Defining what is intended to be obtained in this study begins a process of having a clear outline of how the following sections of data collection and relationship fit together. Collection can be carried out manually, via document review and anonymization, or in an automated way, using data scraping methods and APIs capable of obtaining considerable amounts of data from various sources, allowing researchers to extract valuable insights and identify patterns relevant to the research in question (Josephson et al., 2019). The present research was semi-automated. Table 4 presents the extraction form used.

**Table 4:** Extraction form.

Numeral	Question	Answer
1.	What type of study was carried out?	[Practical Application, Case Study, Proof of Concept, Controlled Experiment]
2.	What are the main objectives of the article?	
3.	What model were investigated in the paper?	[BERT, LLMs]
4.	Does the study have any experimental evaluation?	[Yes, No]
5.	What were the main results obtained in the NER?	
6.	Have threats to validity been declared?	[Yes, No]

### 2.4 Conducting systematic mapping

#### 2.4.1 Protocol execution

1. Access the search databases and conduct the search using the respective search strings;
2. Apply the inclusion filters;
3. Researchers analyze the titles, abstracts, keywords, and methodology, removing works that do not meet the established criteria;
4. Use the blind review method, whose primary objective is to ensure that the evaluation is conducted anonymously, so reviewers do not know the authors’ identities and vice versa. The Rayyan platform will serve as a tool for performing this activity;
5. Evaluate, among peers, if there is a tie in the selection of works, discussing the inclusion or exclusion of the article according to the established criteria;
6. The selected articles will be reviewed to collect metadata for the quality evaluation criteria and any relevant characteristics.

Below, we present Step 1 of the execution protocol for this systematic mapping, along with the results related to the applied inclusion and exclusion criteria.

**Figure 1:**
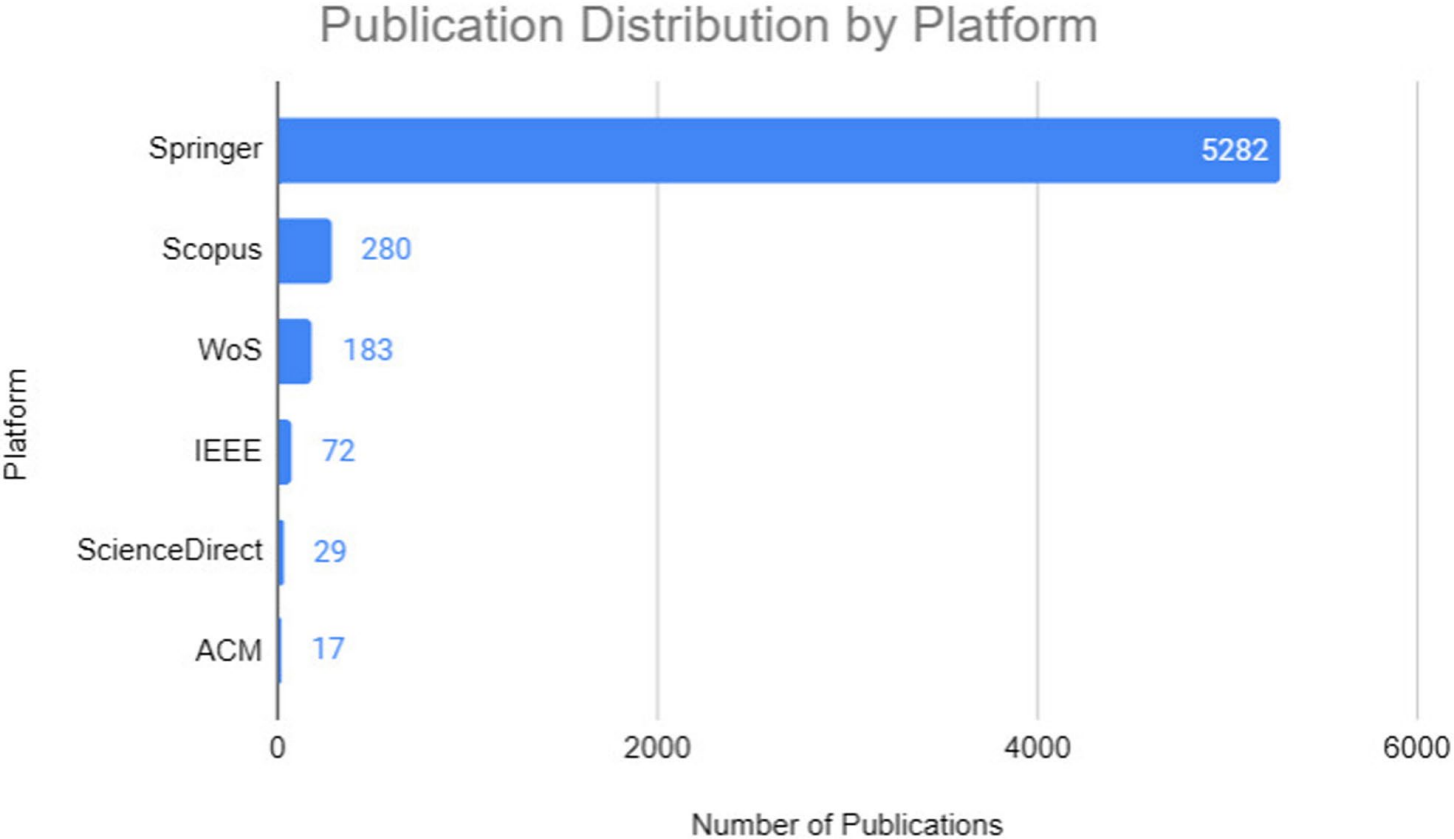
First step of the execution protocol—string search in digital libraries.

**Figure 2:**
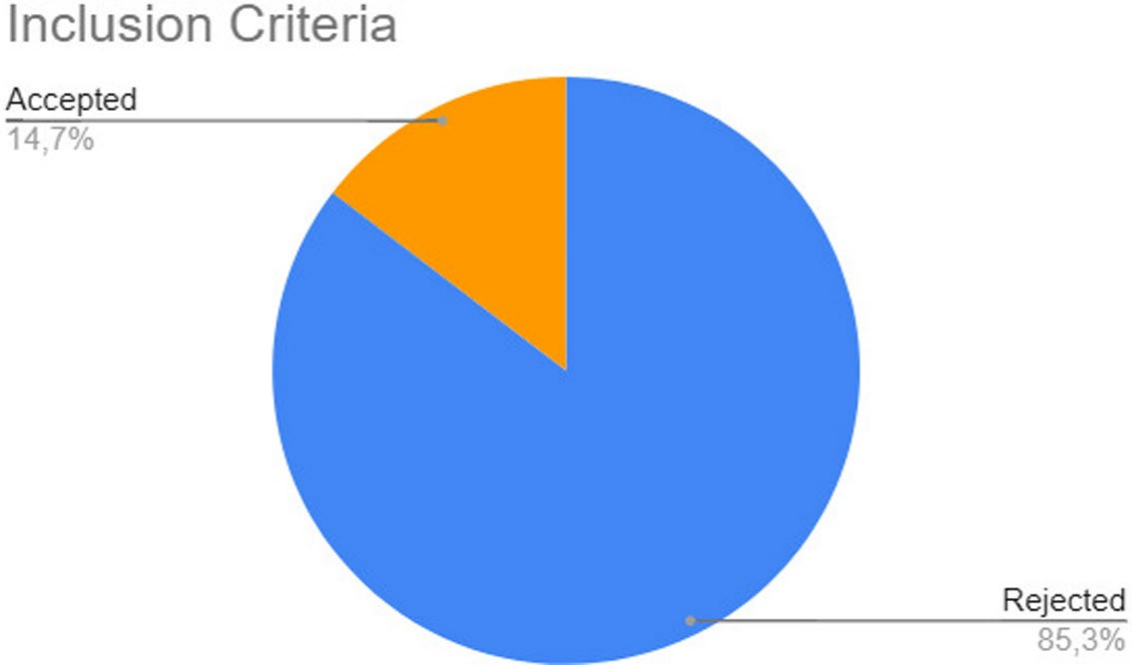
First stage of selection of works.

The results obtained, as shown in Figure 1, were as follows: 72 articles from IEEE Xplore, 280 from Scopus, 29 from ScienceDirect, 5,282 from Springer, 17 from ACM Digital Library, and 183 from Web of Science. These numbers indicate that the Springer database contributed the majority of articles relative to the total, with approximately 90.07%, followed by Scopus with 4.77%, Web of Science with 3.12%, IEEE Xplore with 1.23%, ScienceDirect with 0.49%, and ACM Digital Library with 0.29 (see Figure 2).

**Figure 3:**
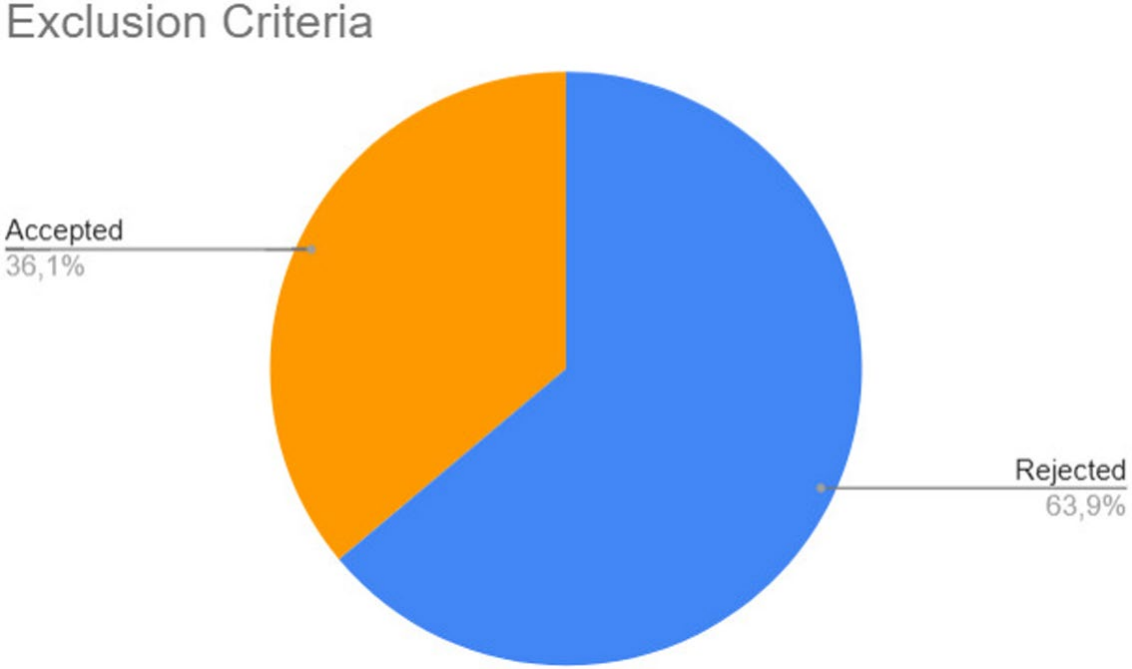
Second stage of selection of works.

After retrieving the articles from the databases, the filtering process began, based on the inclusion criteria defined 2. Each article was classified as Accepted or Rejected. Out of the 5,863 publications analyzed, 5,004 (85%) did not meet the inclusion criteria, inclusion criterion 1 meant that 280 articles remained from Scopus, 29 from Science Direct, 72 from IEEE and 17 from ACM (none were removed), while 4,615 were removed from Springer (leaving 667) and 1 article from Web of Science (leaving 182). Then inclusion criterion 2 meant that 104 articles from Scopus, 25 from Science Direct, 597 from Springer, 2 from IEEE, 130 from Web of Science, and 1 from ACM were returned. After removing these articles, a superficial reading of the remaining works was conducted, analyzing the title, abstract, and keywords. At the end of this stage, 549 articles (64% of the total) were found to be outside the scope of this mapping and were classified as Rejected. Figure 3 provides a summary of this stage. Finally, for detailed analysis, the remaining articles were classified as accepted, where the quality assessment stage will be conducted.

## 3 Result and discussion

**Figure 4:**
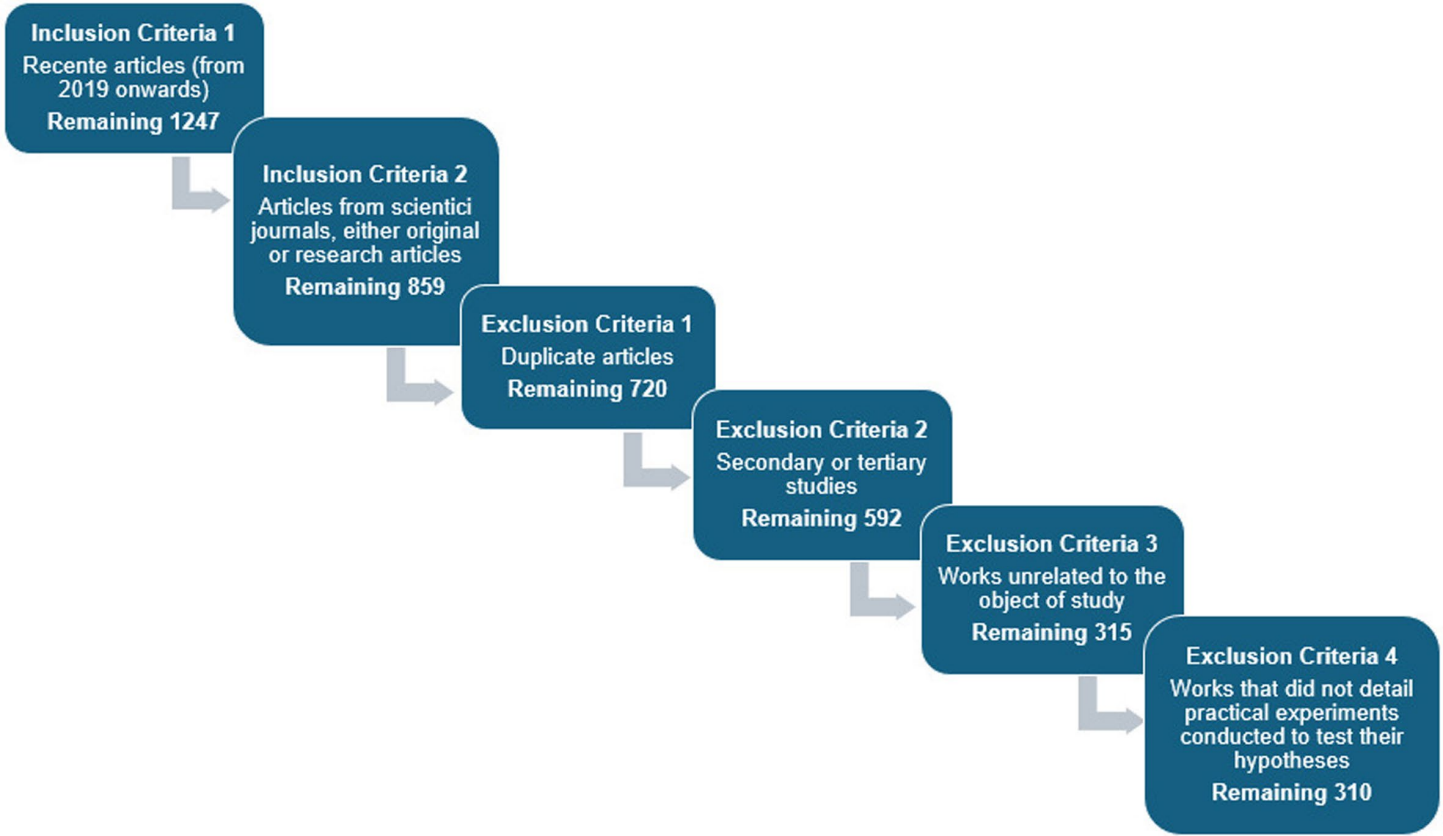
Methodological process (numerical data).

The article collection for the systematic mapping was carried out on April 25, 2024, following established methodological steps. Figure 4 illustrates each step of this process numerically, providing a systematic organization and an essential resource for documenting and clearly communicating the research results.

### 3.1 What are the main techniques used?

**Figure 5:**
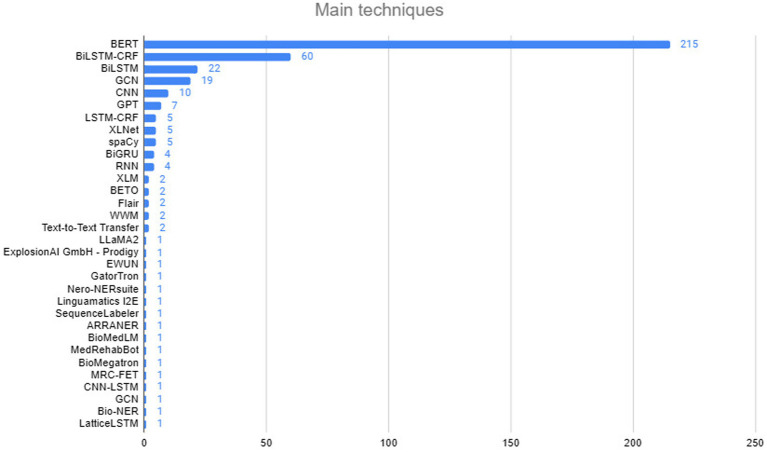
Executing the search string in digital libraries.

Figure 5 presents the main techniques used, including BERT, with 215 occurrences due to its ability to capture the full context of a word in a sequence, making it essential for NLP tasks such as NER. BiLSTM-CRF, with 60 occurrences, combines bidirectional LSTM networks with CRFs for sequence labeling, leveraging context in both directions and capturing dependencies between tags. Other important techniques include BiLSTM (22 occurrences), used in sentiment analysis and text classification, and GCN (19 occurrences), applied to structured data such as social networks. CNN, with 10 occurrences, is also used in NLP, primarily for text classification. Thus, the focus is on deep learning models that capture complex relationships in sequential data.

### 3.2 Which specific techniques perform best and worst (assessment basis language and learning type)?

**Figure 6:**
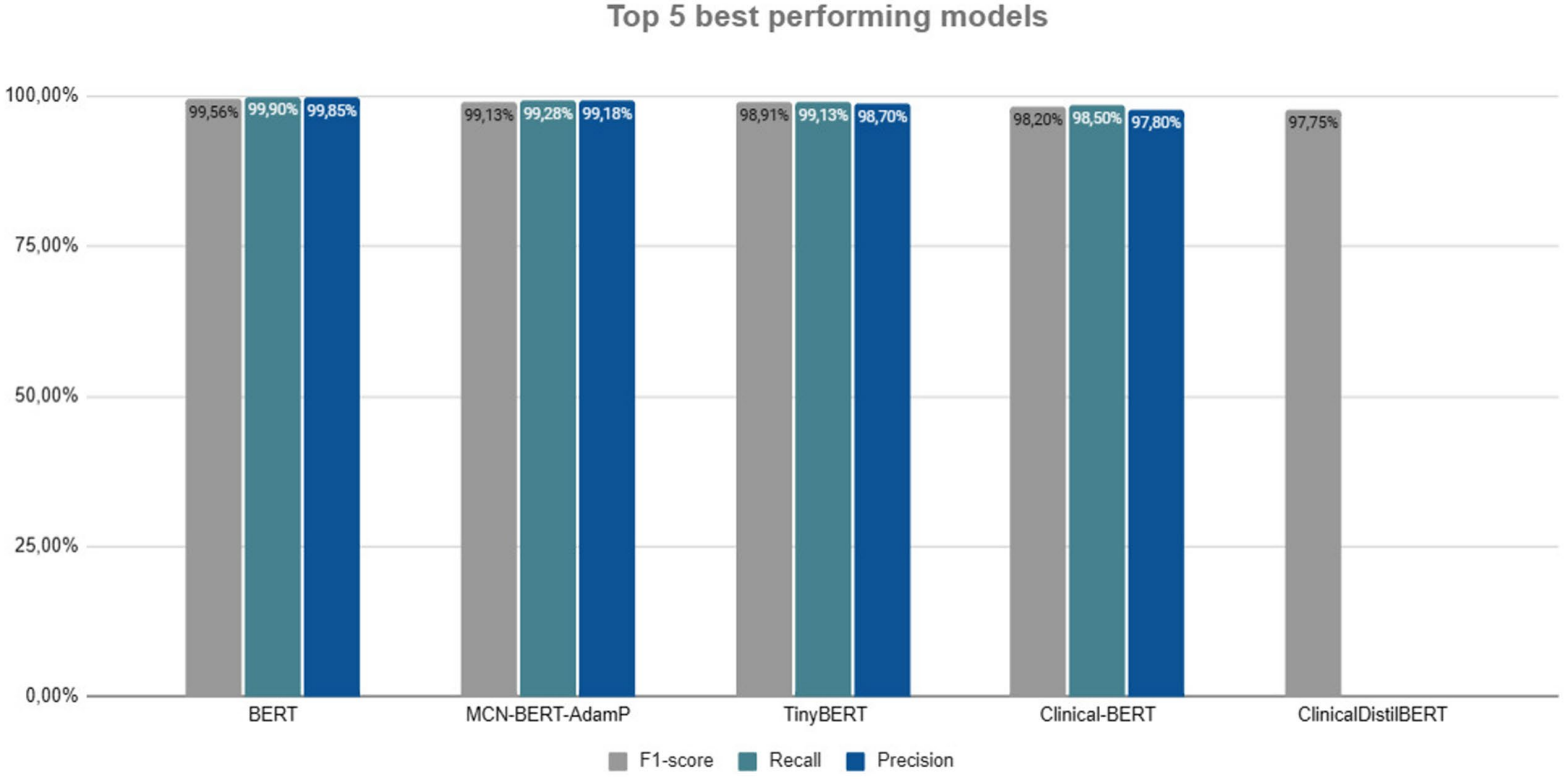
Top 5 best techniques.

The techniques presented and the empirical studies as the most effective for NER in health text using AI, seen in the Figure 6, were identified in the literature by reading the results and discussion of each article, many of these results were in images, requiring a more careful analysis. These studies often also brought results of other activities carried out beyond NER, example sentiment analysis. The comparison between the five best models reveals exceptional performances in terms of F1-score, Recall, and Precision, all above 97%. It is important to note that this study did not compare the NER task across languages, it is possible that the same LLM model may produce different F1-score results (Lee et al., 2024), taking into account the amount, organization, and clarity of data for a specific language.

BERT leads with an F1-score of 99.56%, Recall of 99.90%, and Precision of 99.85%, being the model with the most balanced and consistent performance across all aspects. Following closely, the MCN-BERT-AdamP also shows impressive results with an F1-score of 99.13%, Recall of 99.28%, and Precision of 99.18%. Although slightly inferior to BERT, it maintains a high Precision and Recall, demonstrating excellent robustness. Continuing the analysis, TinyBERT, with an F1-score of 98.91%, Recall of 99.13%, and Precision of 98.70%, offers solid performance but is a bit lower than the top two models. Nonetheless, it remains an efficient alternative, especially in scenarios with computational resource constraints. The Clinical-BERT achieves an F1-score of 98.20%, Recall of 98.50%, and Precision of 97.80%. Although inferior compared to the top three models, it remains an efficient option due to its who has been specifically trained to understand and process medical texts.

Lastly, the ClinicalDistilBERT, with an F1-score of 97.75%, completes the top 5, Recall and Precision were not reported in the study. Its performance is similar to that of the Clinical-BERT, suggesting that both architectures pre-trained specifically with medical data, it has results close to the pure BERT base model, possibly even better.

In Table 5 you can see the language of the assessment base and the type of learning of the 10 most important articles, including those presented above. You will also find the name of the respective article and the evaluation basis used.

**Table 5:** Top 10 best studies and their techniques.

Ranking	F-measure	Article title	Assessment basis	Assessment basis language	Model	Learning type
1	99.56%	Validation of deep learning natural language processing algorithm for keyword extraction from pathology reports in electronic health records.	Pathology reports (Korea University Hospital)	English	BERT	Supervised learning and fine-tuning
2	99.13%	Optimizing classification of diseases through language model analysis of symptoms	Symptom2Disease and Twitter Drug	English	MCN-BERT and BiLSTM	Supervised learning and fine-tuning
3	98.91%	An efficient method for deidentifying protected health information in Chinese electronic health records: algorithm development and validation	EHRs (local hospitals in Chongqing city)	Chinese	TinyBERT	Supervised learning and fine-tuning
4	98.20%	A weakly-supervised named entity recognition machine learning approach for emergency medical services clinical audit	Singapore Civil Defense Force paramedic reports.	English	BERT-base-uncased and Clinical-BERT	Weakly-supervised
5	97.75%	Lightweight transformers for clinical natural language processing	Public pools (MedNLI and i2b2) and an internal pool (ICN)	English	BioDistilBERT, ClinicalDistilBERT and others based on BioClinicalBERT	Supervised learning and fine-tuning
6	96.96%	Automatic de-identification of French electronic health records: a cost-effective approach exploiting distant supervision and deep learning models	EHRs—eHOP CDW Medical Records	French	mBERT, CamemBERT, FlauBERT and Flair + Bi-LSTM-CRF and FastText	Supervised learning
7	96.80%	A Chinese NER model based on BERT with multi knowledge graph fusion and embedding	MSRA-NER and Medical-NER	Chinese	BERT	Supervised learning and fine-tuning
8	96.29%	Research on named entity identification of Tibetan medical ancient books based on hybrid deep learning	The Four Medical Tantras	Chinese	ALBERT and BiLSTM-CRF	Few-shot learning
9	96.27%	An offline English optical character recognition and NER using LSTM and adaptive neuro-fuzzy inference system	EHRs	English	ANFIS-BERT-CRF	Supervised learning and fine-tuning
10	96.27%	A large language model for electronic health records trained from scratch	i2b2 (2010, 2012), n2c2 (2018,2019), MedNLI and emrQA	English	Scaled BERT trained from scratch	Self-supervised pre-training and supervised fine-tuning

**Figure 7:**
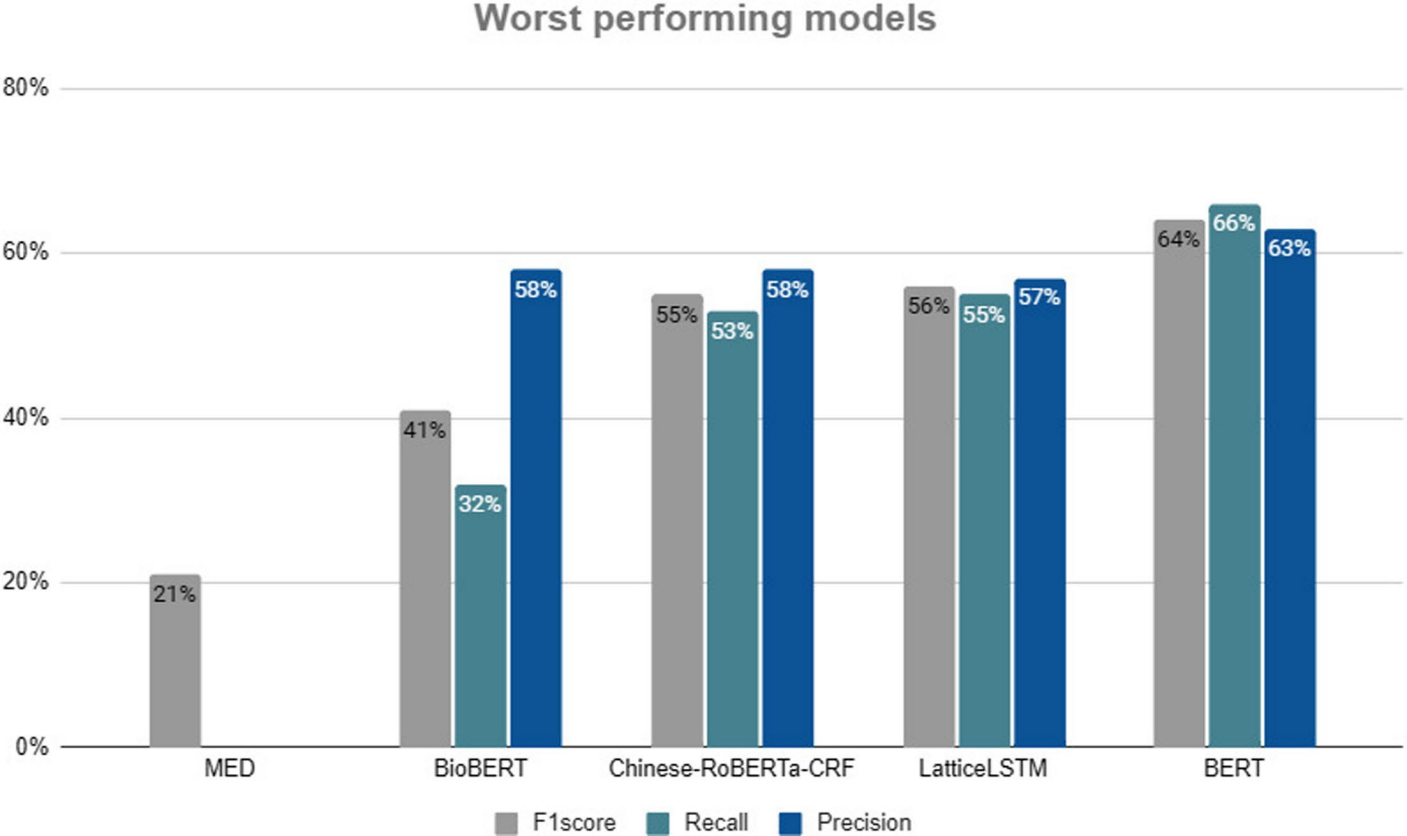
Top 5 worst techniques.

After analyzing the models with the best performance, it is equally important to consider the models with the worst results. Figure 7 shows a summary of the five models that obtained the worst results in each indicator (F1-Score, Recall and Precision). These models are ordered in descending order: the F1-Score was 64%, the Precision was 63.73% and the Recall was 66.25%; as for the least bad model (BERT), the LatticeLSTM model obtained an F1-Score of 56%, a Precision of 57% and a Recall of 55%. Other models were also used in this study, namely the pre-trained BERT and the BiLSTM-CRF. The Chinese ROBERTa-CRF model obtained an F1-score of 55.45%, while the Recall value was lower (55%) and the Precision (58%). Close behind was BioBERT, which obtained an F1-score of 41.30% and in the other two metrics values of 58.10% for Precision and 32.10% for Recall. Finally, the MED model obtained an F1-score of 21.70%, which is the worst performance of all the models identified. Precision and Recall are not reported.

In Table 6 you can see the language of the evaluation base and the type of learning of the 10 articles with the lowest metric returns, including those shown above. You will also find the name of the respective article and the evaluation base used.

**Table 6:** Top 10 worst studies and their techniques.

Ranking	F-measure	Article title	Assessment basis	Assessment basis language	Model	Learning type
1	21.70%	Knowledge grounded medical dialogue generation using augmented graphs	MedDialog(EN) and Covid	English	MED—BioBERT	Supervised learning (fine-tuning)
2	41.30%	Large-scale protein–protein post-translational modification extraction with distant supervision and confidence calibrated BioBERT	Dataset—UMLS IntAct database and PubMed abstracts	English	BioBERT	Distant supervision
3	55.45%	Subsequence and distant supervision based active learning for relation extraction of Chinese medical texts	CMeIE (Chinese Medical Information Extraction)	Chinese	Chinese-RoBERTa-CRF	Active learning
4	56%	A Chinese telemedicine-dialogue dataset annotated for named entities	haodf.com-Chinese telemedicine platform, IMCS-NER and MedDialog-CN	Chinese	BiLSTM-CRF, BERT and LatticeLSTM	Traditional supervised
5	64.97%	A unified knowledge extraction method based on BERT and handshaking tagging	CMeEE	Chinese	BERT	Supervised learning (fine-tuned)
6	68.01%	We are not ready yet: limitations of state-of-the-art disease named entity recognizers	NCBI and BC5CDR	English	BioBERT	Transfer learning (fine-tuning)
7	70%	An evaluation of GPT models for phenotype concept recognition	HPO-GS (Human Phenotype Ontology) and BIOC-GS	English	GPT-3.5-turbo and GPT-4.0	Zero-shot/few-shot learning through in-context learning
8	76%	Machine reading comprehension model in domain-transfer task	NEREL and NEREL-BIO	Russian	RuBERT	Few-shot/zero-shot learning and transfer learning
9	79%	Automated tabulation of clinical trial results: A joint entity and relation extraction approach with transformer-based language representations	Abstracts of scientific articles on RCTs	English	BioBERT, SciBERT and RoBERTa	Few-shot (fine-tuned)
10	91.80%	Survey of transformers and towards ensemble learning using transformers for natural language processing	Tweets, SQuAD 1.1, CNN/Daily Mail, Disaster, Groningen Meaning Bank	English	(BERT, XLNet, RoBERTa, GPT-2 and ALBERT)	Supervised learning (fine-tuned)

By analyzing the tables above, it is possible to see the predominance of the BERT model and its variants in the best results, the English language was the most used language in terms of test and evaluation bases, the most recurrent type of learning is supervised learning with fine-tuning, fine tuning is the adjustment of the pre-trained model to the specific task, which in the case of our study is NER in health texts, we can also see in the table of worst models evaluated that zero-shot and few-shot are used, other types of learning are also more diverse, so we can conclude that using BERT with supervised learning and fine-tuning is considered an option that will certainly bring good results, also taking into account the data set that will be used.

### 3.3 In which years were MOST articles published on using bidirectional pre-training, transformers, or large language models for named entity extraction in health documents?

**Figure 8:**
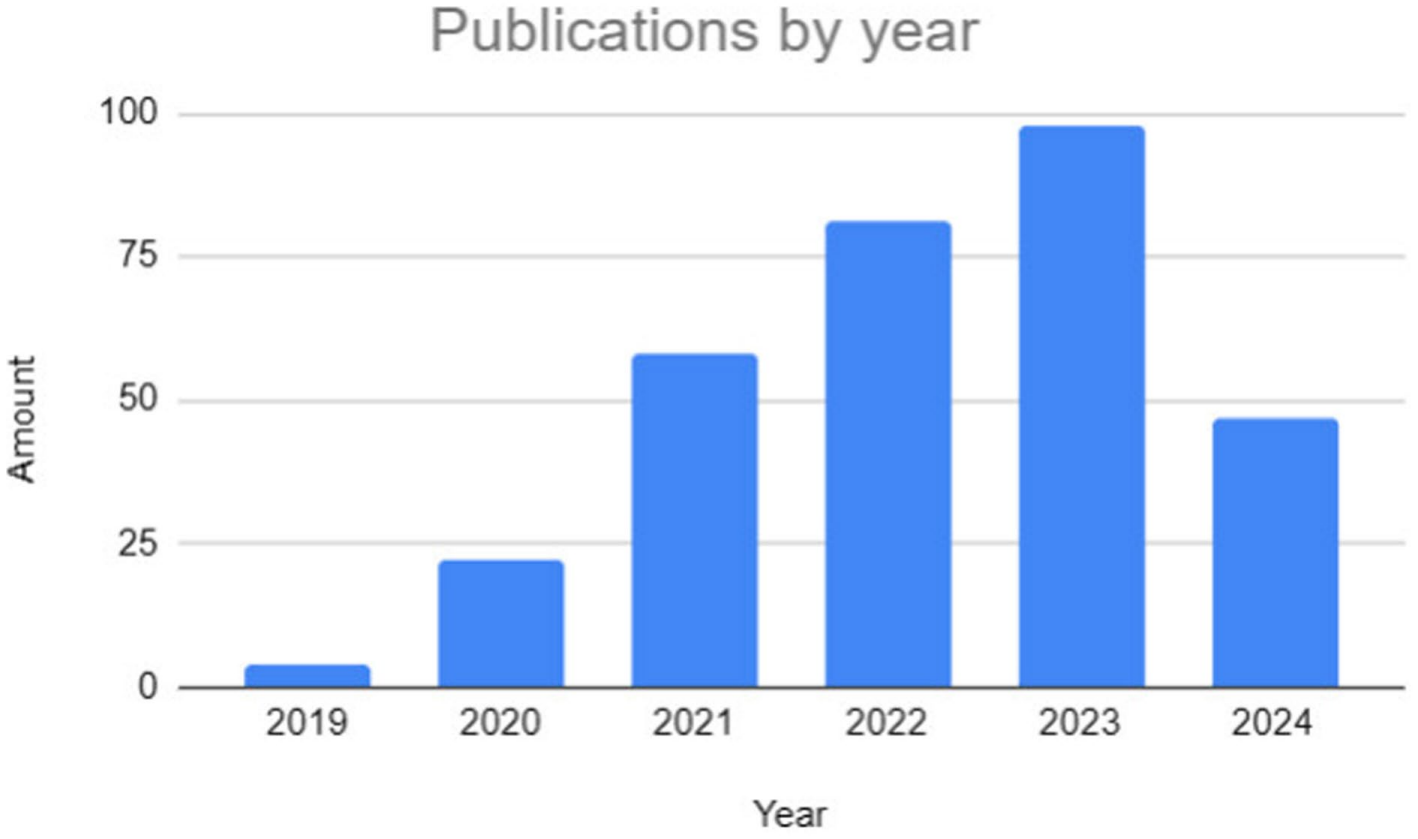
Articles selected by year of publication.

Figure 8 shows the distribution of selected studies by year of publication. It can be seen that most of the studies were published in 2023. BERT was introduced in 2019, and since then there has been a growing interest in using LLMs for NLP tasks. Scientific research usually takes time, and there may be a delay between data collection, analysis, and publication of results. Studies started in 2019 may have taken until 2023 to be completed and published in peer-reviewed journals.

### 3.4 Which countries have contributed the MOST significantly with publications in the context of bidirectional language pre-training techniques, transformers or large language models used in the extraction of named entities in health-related documents?

**Figure 9:**
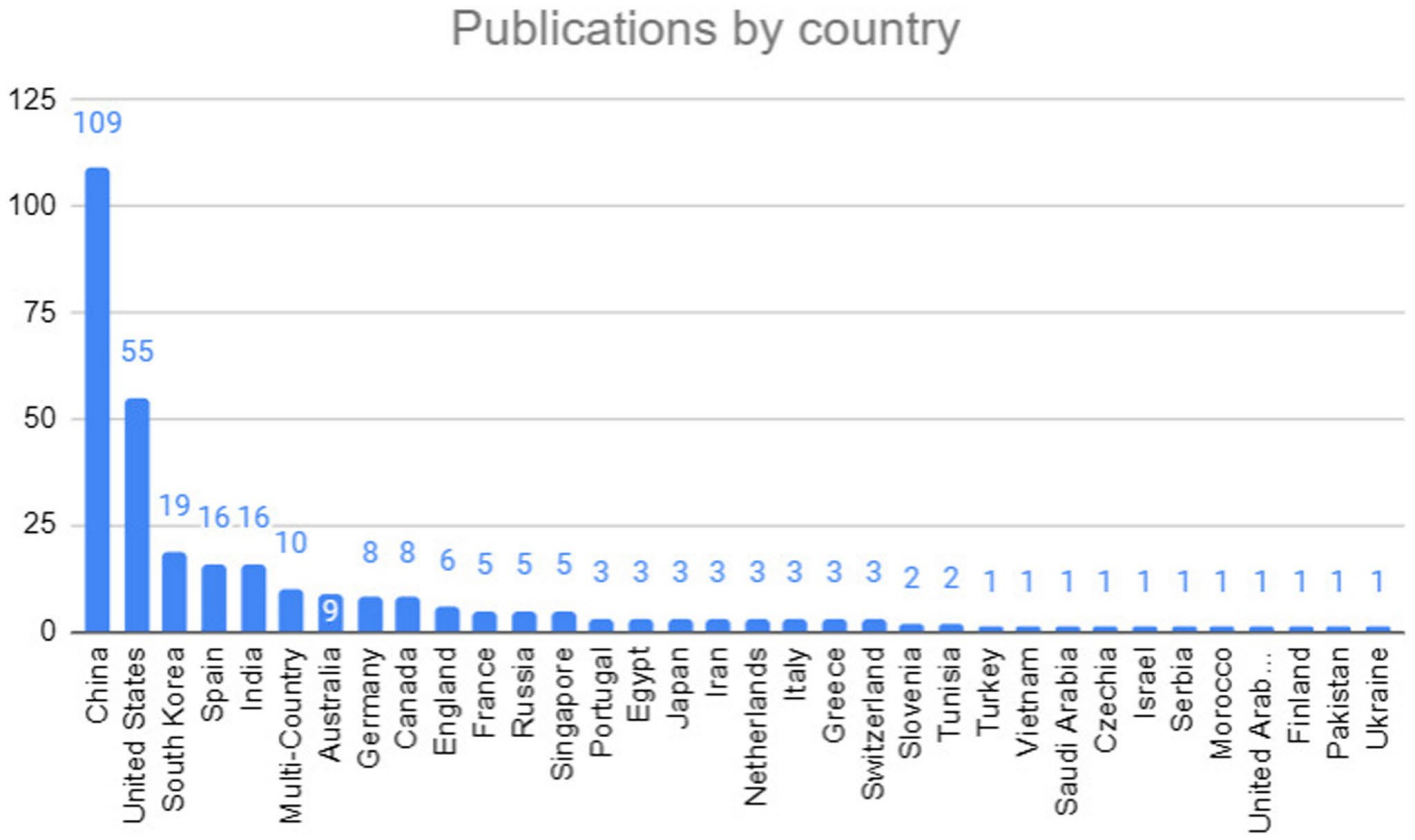
Publications by country.

Figure 9 presents the countries that have published research on the topic addressed in this mapping. The country that stood out the most was China, with the highest number of publications, followed by the United States and South Korea.

## 4 Narrative synthesis

In this section, the key aspects and lessons learned from the analyzed articles will be discussed. Rabinowitz (1987) provides a detailed perspective on how narrative synthesis occurs within a systematic literature mapping. It argues that readers tend to synthesize dispersed elements of a narrative to form a coherent and meaningful understanding of the story. The synthesis focuses on presenting relevant data for the proposed analysis in a summarized manner. In addition to simplifying and making an extensive body of literature accessible, synthesis also helps researchers formulate new questions and identify promising research areas.

Various intelligent approaches and techniques have been applied to NER, with most articles on the topic published in 2023, indicating that this research field is still growing. Furthermore, the results show that publications on this topic span multiple countries, evidencing a global concern for finding effective techniques to assist in the context of health-related texts.

The majority of studies utilize the BERT model and its variants, such as BioBERT, ClinicalBERT, and PubMedBERT, recognized for their high efficacy in NER and Relation Extraction (RE) tasks, often achieving F1-scores above 90%. For instance, one study reported an F1-score of 98.2% (Wang et al., 2021), demonstrating the robust Precision of these models. The variants are fine-tuned for specific domains using data from sources like PubMed, enhancing their understanding and processing of biomedical texts. Another widely used model, BiLSTM-CRF, combines bidirectional recurrent neural networks with CRFs to effectively capture sequential and contextual dependencies, though its performance varies depending on the context and data.

Additionally, hybrid architectures such as BERT-CNN-BiLSTM-CRF (Zhang et al., 2021) integrate different techniques to enhance the capture of context and semantic relationships in the data. These models are evaluated on a variety of corpora, including clinical data (MIMIC-III and i2b2), biomedical data (BC5CDR and NCBI), and social media data. Performance improvements depend on the complexity and type of data, with BioBERT and ClinicalBERT consistently showing advancements in NER and RE tasks. Techniques such as data augmentation and manual annotations also contribute to superior performance.

Innovations like adversarial learning (Guo and Zhang, 2023) and prompt tuning (He et al., 2024) are employed to improve performance on specific tasks. Models such as BioELECTRA and BioALBERT have demonstrated improvements in BioNLP tasks (Naseem et al., 2022), advancing Precision and Recall. Architectures like transformers with attention layers and memory networks are noted for their ability to capture complex relationships within the data. Applied to a wide range of tasks, these models address everything from biomedical entity identification to relation extraction in clinical data, offering significant impact in biomedical research and clinical decision-making.

According to the analyzed data, models like BiLSTM-CRF and BERT-BiLSTM-CRF demonstrate varying efficacy depending on the corpus, with F1-scores fluctuating based on the dataset used. This highlights the importance of fine-tuning and selecting specific models for each task. For example, BiLSTM-CRF (Cheng et al., 2021) achieved an average F1-score of 91.07% on the CCKS2017 and CCKS2018 datasets and 87.05% on the private FCCd dataset. Our analysis suggests that BERT offers slightly higher Precision and solid performance in real-time processing tasks. Specialized variants, such as BioClinicalBERT (Shyr et al., 2024), have also shown superior performance in specific tasks, such as identifying rare diseases and clinical signs, with F1-scores of 0.778 and 0.725, respectively.

## 5 Threats to validity

A threat to the validity of a study is any factor that could affect the internal or external validity of the results obtained. Thus, the study identifies the following risks to validity:

*Selection Bias*: Publications included in this study do not reflect the total population of primary studies over the last five years. Because it is directly related to specific criteria, it does not include the existing diversity of available primary studies.
*Exclusion Bias*: Relevant publications that may have been excluded by the exclusion criteria in this study could lead to underestimation or overestimation of the effects observed.
*Language Bias*: The limitation of studies in the selected language may limit the generalizability of the results to the population or context of another language.
*Time Bias*: The validity of results would not be limited to just the last year, given that practice, technology or the research method would change in substantial ways.

## 6 Final considerations

This study conducted a systematic mapping to explore current limitations, examine emerging technological innovations, and propose strategies for implementing more effective and adaptable solutions to the needs of the modern healthcare environment. Of the 5,863 papers retrieved from scientific databases, 308 met the inclusion and exclusion criteria, with 32% of them published in 2023 alone. This highlights a trend in the field, with growing attention from researchers to the problem in recent years. The primary publication medium selected was journal articles, chosen because they typically undergo a more extensive and detailed peer-review process, resulting in higher quality and reliability of the presented results.

Considering the goal of developing and evaluating an artificial intelligence model focused on NER in health-related news texts from the internet, the techniques and results presented offer a solid foundation for building the proposed model, as mentioned in item 3.2, this study has the limitation of not having tested the same model in different languages. The literature indicates that BERT-based approaches, such as BioBERT, ClinicalBERT, are highly effective for NER and RE, frequently achieving F1-scores above 90%. These variants, tailored to biomedical domains, demonstrate robustness for clinical and public health contexts and should be prioritized for their precision and alignment with our objectives.

Another relevant model, BiLSTM-CRF, while exhibiting variability depending on the data type and corpus, captures sequential and contextual dependencies, which can contribute to accurate entity recognition in news texts. Architectural combinations, such as BERT-CNN-BiLSTM-CRF, along with adversarial learning and techniques like prompt tuning, stand out for integrating different approaches and enhancing NER effectiveness in varied contexts.

For model development, it is essential to explore techniques that enhance the adaptability of NER to health-related texts found in internet news. This includes considering models with greater generalization capabilities, such as GPT and its variants, which have demonstrated superior recall performance and adaptability to new domains—desirable characteristics for maintaining accuracy in a diverse and dynamic textual environment.

In summary, selecting models like BERT and GPT, combined with techniques such as fine-tuning, prompt adaptation, and data augmentation, will enable optimized performance in Precision, Recall, F1-score, and Accuracy metrics, which are essential for the effective identification of health-related entities.

## 7 Future works

Architectural combinations, for example BERT-CNN-BiLSTM-CRF, with adversarial learning and techniques such as fast tuning stand out for integrating different approaches and helping to make NER more effective. For model development, a combination of techniques that have a fast fit will stand out for NER for health text in internet news and they should be explored, i.e., quite “generalist” models such as GPT and variant alternatives popularized by overly positive performance in adaptability to new domains and high Recall, characteristics that are desirable insofar as an optimum level of precision is required in a dynamic and diverse textual context.

In general, the choice of BERT or GPT models along with fine tuning, fast adaptation and data growth will help in optimized performance and higher returns in metrics of precision, recall, F1 score, accuracy, which is essential for identifying health-related entities.

Based on the results of this mapping, a NER experiment will be carried out on health texts. The dataset, comprising approximately 60,000 news items, was previously extracted, pre-processed and classified, as detailed in Fontes et al. (2023).

## Footnotes

^1^ https://www.scopus.com

^2^ https://ieeexplore.ieee.org

^3^ https://dl.acm.org

^4^ https://www.webofscience.com

^5^ https://link.springer.com

^6^ https://www.sciencedirect.com
